# Chronic nicotinamide mononucleotide supplementation elevates blood nicotinamide adenine dinucleotide levels and alters muscle function in healthy older men

**DOI:** 10.1038/s41514-022-00084-z

**Published:** 2022-05-01

**Authors:** Masaki Igarashi, Yoshiko Nakagawa-Nagahama, Masaomi Miura, Kosuke Kashiwabara, Keisuke Yaku, Mika Sawada, Rie Sekine, Yuichiro Fukamizu, Toshiya Sato, Takanobu Sakurai, Jiro Sato, Kenji Ino, Naoto Kubota, Takashi Nakagawa, Takashi Kadowaki, Toshimasa Yamauchi

**Affiliations:** 1 Department of Diabetes & Metabolic Diseases, Graduate School of Medicine, The University of Tokyo, Tokyo, Japan; 2 Data Science Office, Clinical Research Promotion Center, The University of Tokyo Hospital, Tokyo, Japan; 3 Department of Molecular and Medical Pharmacology, Faculty of Medicine, University of Toyama, Toyama, Japan; 4 Department of Clinical Nutrition Therapy, The University of Tokyo Hospital, The University of Tokyo, Tokyo, Japan; 5 Mitsubishi Corporation Life Sciences Limited, Tokyo, Japan; 6 Department of Radiology, Tokyo Metropolitan Police Hospital Tokyo, Tokyo, Japan; 7 Department of Radiation Technology, The University of Tokyo Hospital, Tokyo, Japan; 8 Toranomon Hospital, Tokyo, Japan

**Keywords:** Translational research, Quality of life

## Abstract

Preclinical studies have revealed that the elevation of nicotinamide adenine dinucleotide (NAD + ) upon the administration of nicotinamide mononucleotide (NMN), an NAD + precursor, can mitigate aging-related disorders; however, human data on this are limited. We investigated whether the chronic oral supplementation of NMN can elevate blood NAD + levels and alter physiological dysfunctions in healthy older participants. We administered 250 mg NMN per day to aged men for 6 or 12 weeks in a placebo-controlled, randomized, double-blind, parallel-group trial. Chronic NMN supplementation was well tolerated and caused no significant deleterious effect. Metabolomic analysis of whole blood samples demonstrated that oral NMN supplementation significantly increased the NAD + and NAD + metabolite concentrations. There were nominally significant improvements in gait speed and performance in the left grip test, which should be validated in larger studies; however, NMN exerted no significant effect on body composition. Therefore, chronic oral NMN supplementation can be an efficient NAD + booster for preventing aging-related muscle dysfunctions in humans.

## Introduction

Aging is a risk factor for diabetes, cardiovascular diseases, cancer, and neurological diseases, such as Alzheimer’s disease, and the suppression of physiological decline in aging is an important approach to prevent aging-related diseases^1^.

Aging- and age-related diseases have been shown to be closely related to decreased NAD + levels^2^. In animal studies, the administration of intermediate NAD + metabolites, such as nicotinamide (NAM), nicotinamide mononucleotide (NMN), or nicotinamide riboside (NR), has been shown to increase NAD + concentrations, which helped improve the health and extend the lifespan of the experimental animals^2–6^. Thus, the potential of intermediate NAD + metabolites in improving tissue rejuvenation in humans has led to multiple clinical trials on NR and NMN.

NR, a vitamin B3 analog, is a major vitamin component present in milk (~1 mg/L)^7^. NMN is present in foods such as edamame, broccoli, and meat (~1 mg/100 g food)^8^. However, owing to their extremely low concentrations in foods, it is difficult to obtain these components in sufficient quantity from food. Therefore, purified and concentrated NR and NMN have been used in clinical trials.

The results of NR clinical trials have been reported. In these trials, NR (100–2000 mg/day) was administered to healthy participants or individuals with obesity for a maximum of 12 weeks^9–19^. Most NR clinical trials have reported the safety of NR administration^9–18^ and the elevation of NAD + or NAD + -related metabolites in the blood^9–17^. The most recent report showed that NR increases the fat-free body mass in participants with obesity, although no effect was observed on insulin sensitivity, mitochondrial function, and hepatic and intramyocellular lipid accumulation^17^.

Recently, for the first time, the safety of single-day NMN oral administration was reported in humans^20^. Moreover, while the drafting of this paper was underway, a 10-week, randomized, placebo-controlled, double-blind trial to evaluate the effect of NMN supplementation on metabolic function in 25 postmenopausal women with prediabetes was reported^21^, in which NMN supplementation increased muscle insulin sensitivity, insulin signaling, and remodeling in women with prediabetes who are overweight or obese^21^. Furthermore, the effects of NMN supplementation combined with exercise training have been reported in healthy amateur runners aged 27–50 years^22^. NMN dose-dependently increased the ventilatory threshold and improved aerobic capacity during exercise^22^. However, evidence of the effects of human interventions with NMN remains limited for older adults.

Therefore, to elucidate the safety and efficacy of NMN administration in older adults, we conducted a placebo-controlled, randomized, double-blind, parallel-group study with the administration of 250 mg of NMN to healthy men aged 65 years or more for 12 weeks. We demonstrated that NMN oral supplementation at 250 mg/day in healthy older men for 12 weeks was safe and well-tolerated and significantly increased the levels of NAD + and NAD +-related metabolites in whole blood. Furthermore, NMN administration partly improved muscle performance, evaluated using gait speed and grip strength, in healthy older men. Thus, the chronic oral administration of NMN could be a therapeutic strategy for aging-related disorders in humans, such as sarcopenia.

## Results

### Participant enrollment and baseline characteristics

**Fig. 1:**
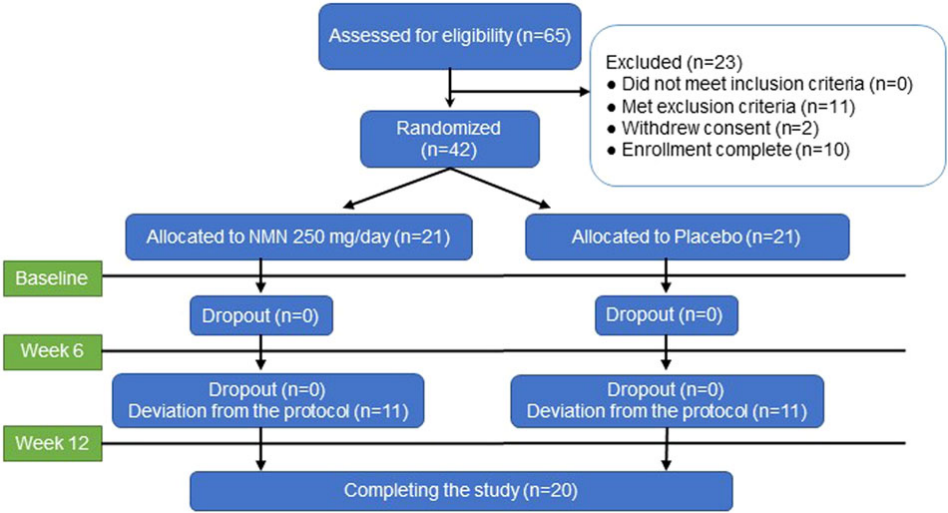
Clinical trial diagrams. Clinical trial flow chart illustrating the procedures for the selection of study participants and data analyses. Sixty-five potential participants were screened, and 42 eligible participants were selected and randomized in a 1:1 ratio into the two groups. Clinical examinations were performed at the 0-, 6-, and 12-week visits. Deviation from the study protocol was noted in 22 participants after the 6-week visit, and eventually, 20 participants completed the 12-week study.

Sixty-five men of age 65 years or more were screened for the study, which was conducted between July 2019 and November 2019 and registered on UMIN-CTR under the identifier UMIN000036321. Eight participants were excluded owing to specific medical history or abnormal laboratory data. Three participants were enrolled in other clinical trials after the obtainment of consent. Two participants were excluded because they requested to withdraw immediately after providing consent. The 42 enrolled participants were randomized between the two treatment groups (placebo group and 250 mg NMN/day group) (Fig. 1).

The supplements (placebo or NMN) were supplied to each group of participants at 0- and 6-week visits. However, after the completion of the study, it came to light that at the 6-week visit, 11 participants each in the NMN and placebo groups received the other supplement owing to an error made by the supplier. According to the decision of the Ethics Committee of the University of Tokyo Hospital, we decided to exclude the data acquired from the 22 participants during the 12-week visit (Fig. 1).

The main physical and metabolic features of the NMN (*n* = 21) and placebo groups (*n* = 21) are summarized in Table 1. Key parameters were comparable between the two groups at baseline. Excluding the data for 22 participants, the physical characteristics of all participants in the NMN and placebo groups at baseline are shown in Supplementary Table S1.

**Table 1:** Clinical characteristics of 42 study participants prior to NMN supplementation.

	Placebo mean ± SD (21)	NMN mean ± SD (21)	Between group *P* value
Age (year)	71.8 ± 6.1	71.1 ± 3.9	0.960^b^
BMI (kg/m^2^)	24.5 ± 1.4	24.1 ± 1.4	0.283^b^
Fat mass (%)	26.7 ± 3.9	25.7 ± 3.8	0.424^a^
SMI (kg/m^2^)	7.62 ± 0.42	7.64 ± 0.29	0.867^a^
Gait speed (m/s)	1.36 ± 0.16	1.45 ± 0.17	0.106^a^
A 30-s chair-stand test (counts/30 s)	14.0 ± 4.2	13.9 ± 3.9	0.909^a^
Right-hand grip strength (kg)	37.7 ± 6.1	39.1 ± 4.7	0.686^b^
Left-hand grip strength (kg)	34.6 ± 4.9	35.1 ± 4.5	0.970^b^
HbA1c (%)	5.82 ± 0.29	5.90 ± 0.53	0.761^b^
FBG (mg/dL)	95.7 ± 10.2	101.0 ± 11.6	0.332^b^
HOMA-IR	1.30 ± 0.83	1.66 ± 1.57	0.406^b^
CT L/S ratio	1.16 ± 0.12	1.14 ± 0.13	0.460^a^
Visceral adipose tissue (cm^2^)	123.0 ± 32.0	124.4 ± 38.7	0.842^b^

The mean and standard deviation of data from the 42 participants (the NMN group (*n* = 21) and the placebo group (*n* = 21)) are presented.

^a^Inter-group comparisons were performed using an unpaired *t* test.

^b^Inter-group comparisons were performed using the Mann–Whitney *U* test.

### Supplementation of 250 mg/day NMN for 12 weeks is well tolerated

We observed excellent adherence to the study treatment, with all participants consuming more than 90% of all NMN and placebo supplements administered. NMN (250 mg/day) was well-tolerated, and no serious adverse event occurred. Clinical laboratory values were obtained from blood samples collected at baseline and at the 12-week visit. No significant difference was observed between the NMN and placebo groups with respect to hematological and blood chemistry parameters, including liver enzymes and renal function markers (Supplementary Tables S2 and S3). Importantly, all clinical laboratory values were within the normal range in the NMN group. These results indicated that NMN supplementation at 250 mg/day for 12 weeks is well tolerated in healthy older men.

### Chronic oral administration of NMN increases the levels of NAD+ and NAD+-related metabolites in whole blood

**Fig. 2:**
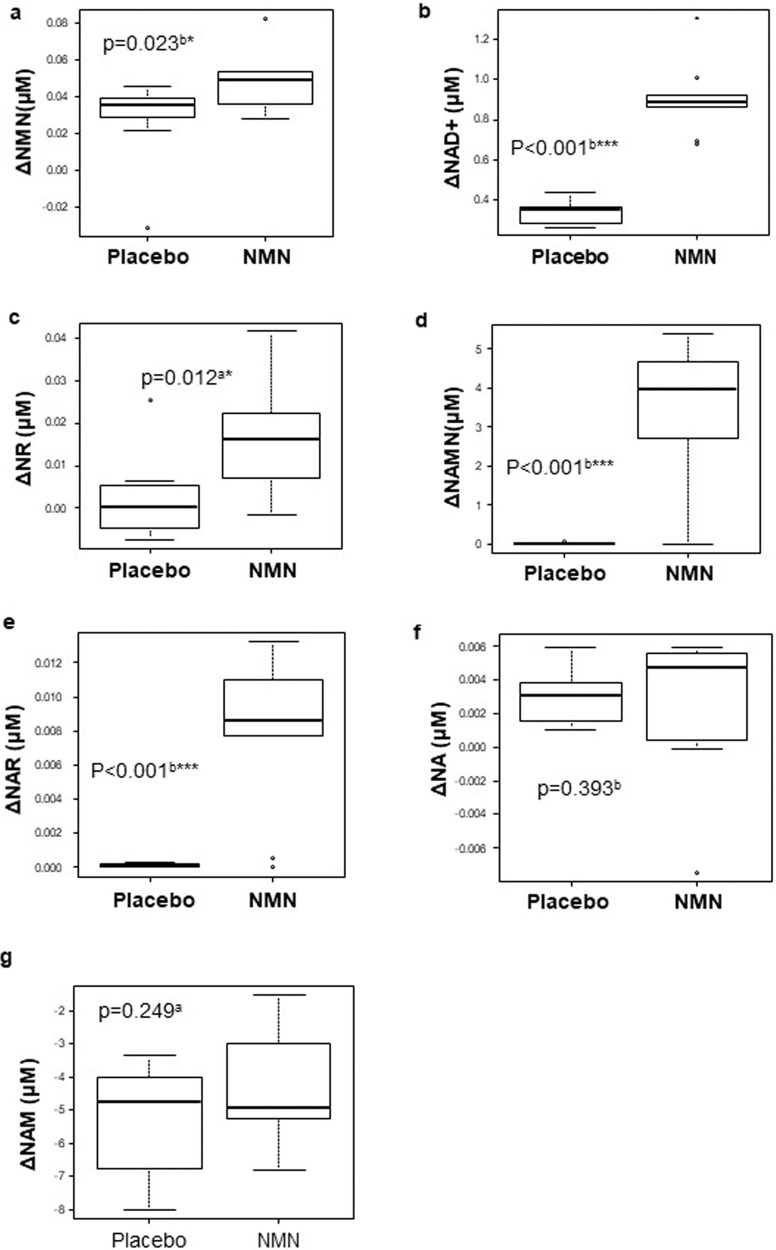
Chronic oral NMN administration increases whole blood NAD+ and NAD+-related metabolite levels. **a**–**g** Changes in whole blood NAD+ and NAD+-related metabolite levels (NMN (**a**), NAD+ (**b**), NR (**c**), NAMN (**d**), NAR (**e**), NA (**f**), and NAM (**g**)) after 12 weeks of placebo (*n* = 10) or NMN (*n* = 10) supplementation. Center lines in boxplots are medians. Bounds of boxes are 25- and 75 percentiles. Bounds of whiskers are the minimum and the maximum inside a distance of 1.5 times the interquartile range from the lower or upper quartile. NMN nicotinamide mononucleotide, NAD+ nicotinamide adenine dinucleotide, NR nicotinamide riboside, NAMN nicotinic acid mononucleotide, NAR nicotinic acid riboside, NA nicotinic acid, NAM nicotinamide. ^a^Inter-group comparisons were performed using an unpaired *t* test. ^b^Inter-group comparisons were performed using the Mann–Whitney *U* test. *P < 0.05; **P < 0.01; ***P < 0.001.

Whole blood samples were collected at baseline and at the 12-week visit from participants for the subsequent analysis of NAD+ and NAD+-related metabolites using liquid chromatography-tandem mass spectrometry (LC-MS/MS). Oral NMN supplementation effectively elevated the levels of NMN and NAD+ as compared to placebo supplementation (Fig. 2 and Supplementary Table S4). We also observed an increase in NR, which could indicate the possible conversion of NMN to NR by CD73^23^. Notably, NMN also significantly elevated the levels of nicotinic acid mononucleotide (NAMN) (an intermediate of the NAD+ de novo synthesis pathway) and nicotinic acid riboside (NAR). Collectively, these findings indicate that the chronic oral supplementation of NMN effectively stimulates NAD+ metabolism in healthy older men.

### Chronic oral administration of NMN partly improves motor functions

To examine the effects of NMN oral administration on skeletal muscle mass in healthy older men, the skeletal mass index (SMI) and segmental lean mass (lean trunk, arms, and legs) were measured using bioimpedance analysis (BIA), and as the primary analysis, the mean values in the NMN and placebo groups at baseline and the 6- and 12-week visits were evaluated using mixed-model analysis or the mixed-effect model for repeated measures (MMRM)^24^. In addition, the means of each group at both visits were compared using the Mann–Whitney *U* test for non-normal distribution and *t* tests for normal distribution. Furthermore, the difference between pre- and post-placebo and pre- and post-NMN supplementation (ΔPlacebo and ΔNMN, respectively) groups at the 6-and 12-week visits were analyzed using ANCOVA. No significant difference was observed in the skeletal muscle mass in any of these analyses (Table 2).

**Table 2:** Effect of NMN on muscle mass.

	Placebo mean ± SD	NMN mean ± SD	*P* value		Placebo mean ± SD	NMN mean ± SD	*P* value
SMI (kg/m^2^) *P* = 0.979^a^, *P* = 0.874^b^				Segmental lean/trunk (kg) *P* = 0.094^a^, *P* = 0.605^b^			
Baseline	7.62 ± 0.42 (21) 7.79 ± 0.44 (10)	7.64 ± 0.29 (21) 7.65 ± 0.39 (10)	0.867^c^ (21:21) 0.459^c^ (10:10)	Baseline	21.2 ± 1.97 (21) 21.3 ± 2.37 (10)	22.2 ± 1.51 (21) 22.2 ± 1.85 (10)	0.076^c^ (21:21) 0.357^c^ (10:10)
Week 6	7.69 ± 0. 40 (21) 7.85 ± 0.42 (10)	7.64 ± 0.32 (21) 7.65 ± 0.40 (10)	0.703^c^ (21:21) 0.253^d^ (10:10)	Week 6	21.2 ± 1.94 (21) 21.5 ± 2.30 (10)	21.9 ± 1.47 (21) 21.9 ± 1.78 (10)	0.113^d^ (21:21) 0.669^c^ (10:10)
Week 12	7.64 ± 0.52 (10)	7.52 ± 0.43 (10)	0.582^c^ (10:10)	Week 12	21.1 ± 2.25 (10)	21.9 ± 1.89 (10)	0.412^c^ (10:10)
Change from baseline to week 6	0.06 ± 0.15 (21) 0.06 ± 0.12 (10)	0.00 ± 0.13 (21) 0.00 ± 0.13 (10)	0.167^e^ (21:21) 0.267^e^ (10:10)	Change from baseline to week 6	−0.1 ± 0.53 (21) 0.2 ± 0.47 (10)	−0.3 ± 0.41 (21) −0.3 ± 0.41 (10)	0.328^e^ (21:21) 0.039^e^* (10:10)
Change from baseline to week 12	−0.15 ± 0.22 (10)	−0.13 ± 0.21 (10)	0.805^e^ (10:10)	Change from baseline to week 12	−0.2 ± 0.37 (10)	−0.3 ± 0.51 (10)	0.706^e^ (10:10)
Segmental lean/right arm (kg) *P* = 0.374^b^				Segmental lean/right leg (kg) *P* = 0.268^a^, *P* = 0.702^b^			
Baseline	2.54 ± 0.32 (21) 2.54 ± 0.35 (10)	2.68 ± 0.24 (21) 2.67 ± 0.28 (10)	0.120^c^ (21:21) 0.364^c^ (10:10)	Baseline	7.92 ± 0.81 (21) 8.10 ± 0.90 (10)	8.16 ± 0.58 (21) 8.21 ± 0.72 (10)	0.274^c^ (21:21) 0.769^c^ (10:10)
Week 6	2.53 ± 0.30 (21) 2.57 ± 0.34 (10)	2.62 ± 0.23 (21) 2.60 ± 0.27 (10)	0.345^d^ (21:21) 0.852^c^ (10:10)	Week 6	8.05 ± 0.81 (21) 8.15 ± 0.93 (10)	8.27 ± 0.57 (21) 8.36 ± 0.70 (10)	0.332^c^ (21:21) 0.577^c^ (10:10)
Week 12	2.49 ± 0.35 (10)	2.60 ± 0.28 (10)	0.443^c^ (10:10)	Week 12	7.91 ± 0.94 (10)	8.15 ± 0.70 (10)	1.000^d^ (10:10)
Change from baseline to week 6	−0.01 ± 0.10 (21) 0.04 ± 0.08 (10)	−0.05 ± 0.08 (21) −0.07 ± 0.07 (10)	0.283^e^ (21:21) 0.012^e^* (10:10)	Change from baseline to week 6	0.14 ± 0.23 (21) 0.05 ± 0.20 (10)	0.11 ± 0.19 (21) 0.15 ± 0.19 (10)	0.804^e^ (21:21) 0.275^e^ (10:10)
Change from baseline to week 12	−0.05 ± 0.08 (10)	−0.07 ± 0.09 (10)	0.667^e^ (10:10)	Change from baseline to week 12	−0.19 ± 0.36 (10)	−0.05 ± 0.31 (10)	0.367^e^ (10:10)
Segmental lean/left arm (kg) *P* = 0.761^b^				Segmental lean/left leg (kg) *P* = 0.251^a^, *P* = 0.891^b^			
Baseline	2.51 ± 0.33 (21) 2.54 ± 0.41 (10)	2.67 ± 0.25 (21) 2.66 ± 0.32(10)	0.081^c^ (21:21) 0.462^c^ (10:10)	Baseline	7.78 ± 0.82 (21) 8.02 ± 0.93 (10)	8.02 ± 0.50 (21) 8.05 ± 0.59 (10)	0.264^c^ (21:21) 0.925^c^ (10:10)
Week 6	2.49 ± 0.32 (21) 2.57 ± 0.39 (10)	2.61 ± 0.24 (21) 2.60 ± 0.30 (10)	0.183^c^ (21:21) 0.854^c^ (10:10)	Week 6	7.84 ± 0.77 (21) 8.02 ± 0.86 (10)	8.07 ± 0.53 (21) 8.12 ± 0.62 (10)	0.256^c^ (21:21) 0.768^c^ (10:10)
Week 12	2.50 ± 0.39 (10)	2.60 ± 0.33 (10)	0.538^c^ (10:10)	Week 12	7.89 ± 1.03 (10)	7.92 ± 0.60 (10)	0.923^c^ (10:10)
Change from baseline to week 6	−0.01 ± 0.11 (21) 0.03 ± 0.09 (10)	0.06 ± 0.09 (21) −0.06 ± 0.09 (10)	0.351^e^ (21:21) 0.0548^e^ (10:10)	Change from baseline to week 6	0.06 ± 0.23 (21) 0.00 ± 0.25 (10)	0.06 ± 0.17 (21) 0.07 ± 0.17 (10)	0.818^e^ (21:21) 0.473^e^ (10:10)
Change from baseline to week 12	−0.04 ± 0.06 (10)	−0.06 ± 0.11 (10)	0.695^e^ (10:10)	Change from baseline to week 12	−0.13 ± 0.33 (10)	−0.13 ± 0.24 (10)	0.979^e^ (10:10)

The numbers in the parentheses indicate the respective sample sizes.

^a^Treatment was compared using a mixed-model analysis. The *P* value denotes interaction.

^b^Treatment was compared using MMRM. The *P* value denotes interaction.

^c^Inter-group comparisons were performed using an unpaired *t* test (no adjustment for baseline).

^d^Inter-group comparisons were performed using the Mann–Whitney *U* test (no adjustment for baseline).

^e^Inter-group comparisons were performed using ANCOVA for adjusting the baseline.

Conversely, to examine muscle strength and performance, gait speed, counts in the 30-s chair-stand test, and grip strength were assessed and analyzed using the same statistical method (Table 3). Mixed-model analysis or MMRM showed a significant improvement in the gait speed (*P* = 0.033) and left grip test (*P* = 0.019) after NMN administration (Table 3). These findings indicate that the chronic oral supplementation of NMN partly improved the muscle strength and performance in healthy older men, although NMN did not affect the skeletal muscle mass.

**Table 3:** NMN partly improves muscle strength and performance.

	Placebo mean ± SD	NMN mean ± SD	*P* value		Placebo mean ± SD	NMN mean ±S D	*P* value
Gait speed (m/s) *P* = 0.033^a^*, *P* = 0.015^b^*				Right grip strength (kg) *P* = 0.259^a^, *P* = 0.194^b^			
Baseline	1.36 ± 0.16 (21) 1.31 ± 0.19 (10)	1.45 ± 0.17 (21) 1.50 ± 0.20 (10)	0.106^c^ (21:21) 0.041^c^* (10:10)	Baseline	37.7 ± 6.1 (21) 36.0 ± 7.2 (10)	39.1 ± 4.7 (21) 40.1 ± 3.3 (10)	0.686^d^ (21:21) 0.147^d^ (10:10)
Week 6	1.34 ± 0.18 (21) 1.27 ± 0.18 (10)	1.47 ± 0.16 (21) 1.54 ± 0.17 (10)	0.023^c^* (21:21) 0.004^c^** (10:10)	Week 6	39.2 ± 5.3 (21) 38.7 ± 6.3 (10)	40.6 ± 4.0 (21) 41.8 ± 4.7 (10)	0.348^c^ (21:21) 0.230^c^ (10:10)
Week 12	1.30 ± 0.22 (10)	1.60 ± 0.13 (10)	0.002^c^** (10:10)	Week 12	37.3 ± 5.9 (10)	41.9 ± 5.6 (10)	0.090^c^. (10:10)
Change from baseline to week 6	−0.02 ± 0.13 (21) −0.04 ± 0.11 (10)	0.02 ± 0.09 (21) 0.03 ± 0.10 (10)	0.111^e^ (21:21) 0.030^e^* (10:10)	Change from baseline to week 6	1.6 ± 3.4 (21) 2.7 ± 3.3 (10)	1.5 ± 4.0 (21) 1.7 ± 4.6 (10)	0.669^e^ (21:21) 0.974^e^ (10:10)
Change from baseline to week 12	−0.01 ± 0.10 (10)	0.09 ± 0.13 (10)	0.066^c^ (10:10)	Change from baseline to week 12	1.3 ± 3.2 (10)	1.8 ± 4.2 (10)	0.479^e^ (10:10)
A 30-s chair-stand test (counts/30 s) *P* = 0.509^a^, *P* = 0.309^b^				Left grip strength (kg) *P* = 0.019^b^*			
Baseline	14.0 ± 4.2 (21) 13.5 ± 5.2 (10)	13.9 ± 3.9 (21) 14.8 ± 4.0 (10)	0.909^c^ (21:21) 0.539^c^ (10:10)	Baseline	34.6 ± 4.9 (21) 34.8 ± 4.8 (10)	35.1 ± 4.5 (21) 36.1 ± 5.7 (10)	0.970^d^ (21:21) 0.587^c^ (10:10)
Week 6	13.6 ± 4.4 (21) 13.7 ± 5.0 (10)	15.1 ± 4.2 (21) 16.1 ± 4.6 (10)	0.261^c^ (21:21) 0.278^c^ (10:10)	Week 6	34.7 ± 5.0 (21) 35.5 ± 5.0 (10)	36.6 ± 5.4 (21) 37.0 ± 6.2 (10)	0.207^d^ (21:21) 0.402^d^ (10:10)
Week 12	14.0 ± 5.2 (10)	16.3 ± 3.6 (10)	0.267^c^ (10:10)	Week 12	34.1 ± 4.6 (10)	37.4 ± 5.8 (10)	0.177^c^ (10:10)
Change from baseline to week 6	−0.5 ± 2.6 (21) 0.2 ± 2.3 (10)	1.2 ± 2.1 (21) 1.3 ± 1.6 (10)	0.031^e^* (21:21) 0.214^e^ (10:10)	Change from baseline to week 6	0.1 ± 3.4 (21) 0.7 ± 1.7 (10)	1.5 ± 4.3 (21) 0.9 ± 4.5 (10)	0.204^e^ (21:21) 0.815^e^ (10:10)
Change from baseline to week 12	0.5 ± 3.7 (10)	1.5 ± 1.7 (10)	0.311^e^ (10:10)	Change from baseline to week 12	−0.7 ± 2.5 (10)	1.3 ± 2.7 (10)	0.082^e^. (10:10)

The numbers in the parentheses indicate the respective sample sizes.

^a^Treatments were compared using a mixed-model analysis. The *P* value denotes interaction.

^b^Treatments were compared using MMRM. The *P* value denotes interaction.

^c^Inter-group comparisons were performed using an unpaired *t* test (no adjustment for baseline).

^d^Inter-group comparisons were performed using the Mann–Whitney *U* test (no adjustment for baseline).

^e^Inter-group comparisons were performed using ANCOVA for adjusting the baseline.

*P < 0.05; **P < 0.01; ***P < 0.001.

We also observed a significant difference between the mean gait speed values of each group during the 6-and 12-week visits (*P* = 0.023 and *P* = 0.002, respectively). Furthermore, a significant difference was observed in the results of the 30-second chair-stand test between the ΔPlacebo and ΔNMN groups at the 6-week visit (*P* = 0.031).

### Liver and visceral fat mass are not affected by NMN supplementation

**Fig. 3:**
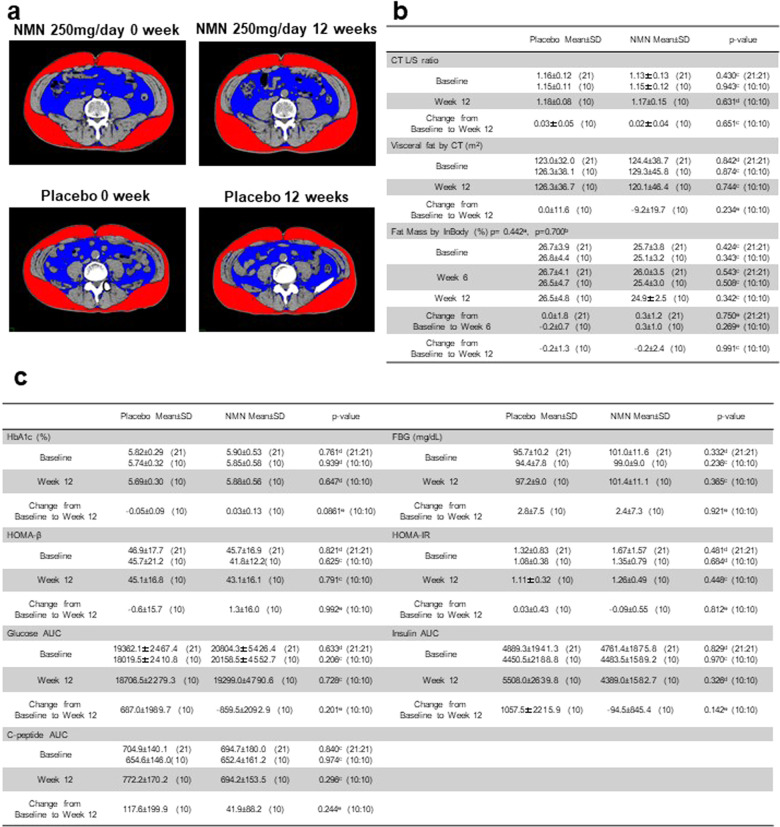
NMN supplementation does not affect metabolic parameters. **a** A representative single CT slice at the navel level of an NMN or placebo group participant at the 0- or 12-visit to calculate the visceral fat area; the red region indicates visceral fat, and the blue region indicates subcutaneous fat. **b** The effect of NMN on the CT L/S ratio, visceral fat area calculated from CT slices, and fat mass (lrb%) measured using the BIA method. **c** The effect of NMN on HbA1c, FBG, HOMA-β, HOMA-IR, glucose AUC, insulin AUC, and C-peptide AUC. The AUC was calculated from the results of the 75 g OGTT. The numbers in the parentheses indicate the respective sample sizes. ^a^Treatment was compared using a mixed-model analysis. The p-value denotes the interaction. ^b^Treatment was compared using MMRM. The *P* value denotes interaction. ^c^Inter-group comparisons were performed using an unpaired *t* test (no adjustment for baseline). ^d^Inter-group comparisons were performed using the Mann–Whitney *U* test (no adjustment for baseline). ^e^Inter-group comparisons were performed using ANCOVA for adjusting the baseline. *P < 0.05; **P < 0.01; ***P < 0.001.

Next, we investigated the effect of NMN on fat mass distribution, because findings from animal studies suggest the positive effect of NMN on insulin sensitivity and hepatic steatosis^3,4^ (Fig. 3). Chronic NMN supplementation did not affect the visceral fat area (Fig. 3a) and CT values of the liver and spleen (L/S ratio) in the computed tomography (CT) scan (Fig. 3b), in accordance with the measurement of fat mass using BIA (Fig. 3b). Likewise, NMN administration did not affect the homeostatic model assessment of insulin resistance (HOMA-IR), an indicator of hepatic insulin sensitivity in blood analysis (Fig. 3c). Adiponectin and interleukin (IL) 6, which are also related to insulin sensitivity, were unaffected by NMN administration (Supplementary Table S5). These data indicate that insulin sensitivity and fat mass were unaffected by NMN supplementation in our study. Consistently, no significant difference or trend of change was observed in the triglyceride, LDL-cholesterol, HDL-cholesterol, HbA1c, FBG, HOMA-β, insulin, and C-peptide levels and the area under the curve (AUC) for glucose in 75 g of the sample using the oral glucose tolerance test (OGTT) after NMN supplementation (Fig. 3c and Supplementary Table S5).

### Effect of NMN on other aging-related phenotypes

To gain exploratory insights into the potential benefits of NMN supplementation on other domains of physiological functions in healthy older men, we assessed a wide variety of outcomes indicative of sensory, vascular, and cognitive functions. Audibility tended to improve in the right ear, although the change was not statistically significant (*P* = 0.054, mixed-model analysis) (Supplementary Table S6). Conversely, no difference was observed in the indicators of vascular functions, such as assessed blood pressure and flow-mediated dilation (Supplementary Table S7). Lastly, the intervention exerted no observable effect on overall cognitive function, as assessed using the mini-mental state examination-Japanese (MMSE-J) and the Japanese version of the Montreal Cognitive Assessment (MOCA-J) (Supplementary Table S6).

## Discussion

In this study, we reported that the chronic oral supplementation of 250 mg of NMN per day is safe and a well-tolerated and effective strategy for boosting NAD+ metabolism in healthy older men. In addition, our exploratory analyses of the effects of NMN supplementation on physiological functions suggest NMN improves muscle strength, which is an important clinical indicator of aging.

When this study was designed, the results of the NMN clinical trial were not available. Some studies have reported the effects of oral or intraperitoneal NMN administration (100–500 mg/kg/day) in mice^3,4^. Particularly, the long-term administration of NMN at doses of 100 or 300 mg/kg/day for 1 year caused no significant side effect and improved insulin sensitivity and eye functions^8^. If this dosage is expressed in terms of the absorption area in the small intestine, 100 mg/kg/day NMN in mice is considered equivalent to an intake of 8 mg/kg/day in humans^25^. Some human clinical studies have been conducted in Japan and the USA. In the study performed in the USA^21^, NMN was administered at 250 mg/day for 10 weeks, and in the study performed in Japan (UMIN ID UMIN000025739), it was administered at 100 or 200 mg/day for 24 weeks. Additionally, several human clinical trials have been conducted on NR, another precursor of NAD+, in which NR has been administered at doses of 100–2000 mg/day for up to 12 weeks, with no serious side effects reported^9–19^. The NMN dose in this study was fixed after considering the dose in previously reported NR trials (100–2000 mg/day) and ongoing NMN clinical trials (100, 200, and 250 mg/day).

In a previous NMN study, a single oral dose of 500 mg NMN^20^ and the 10-week chronic administration of 250 mg NMN^21^ did not induce any specific deleterious side effects. In the most recent study, the chronic administration of 1200 mg of NMN per day for 6 weeks did not cause any significant adverse events^22^. Likewise, the 12-week chronic administration of 250 mg of NMN in this study also exerted no significant side effect. NR has been demonstrated to be well-tolerated in all published clinical studies^9–19^, whereas nicotinic acid (NA) has been demonstrated to induce nausea and flushing, which leads to difficulties in the administration of high doses of NA for increasing NAD+ levels^26^. NAM, another NAD+ precursor, has also been reported to induce hepatotoxicity^27^; however, we did not find any abnormality in the clinical laboratory values, including those of liver or muscle enzymes, in our study. Overall, NMN was well tolerated up to a chronic dose of 250 mg.

Previous studies have reported that NR administration significantly increases the plasma or whole blood NAD+ levels in healthy participants^9–12,15,16^. Conversely, a recent NMN study^21^ showed the direct detection of an increase in the blood NAD+ levels, although the muscle NAD+ content remained unaltered, after 10 weeks of treatment^21^. Thus, this is the second study to report that NMN administration significantly increased the levels of NAD+ and NAD+ metabolites in whole blood. An unexpected finding was a considerable elevation in the NAMN and NAR levels, which was not an en route for the conversion of NMN to NAD+. The previous report has proposed that as the rate of NAD+ synthesis increases, the deamidation of NAD+ to nicotinic acid adenine dinucleotide (NAAD) can occur in competition with NAD+ turnover to nicotinamide, suggesting that NAAD can serve as a sensitive biomarker for increased NAD+ metabolism^9^. Alternatively, an increase in NAD+ can result in NMN deamidation, leading to the formation of NAMN. Another mechanism could be the deamidation of NMN by gut microbiota. Oral NAM or NR can be deamidated into NA, NAR, NAAD, and NAMN by gut microbiota in the small intestine and colon^28^. Deamidated NAD+ metabolites move to the tissues via circulation, contributing to NAD+ synthesis^28^.

Skeletal muscle mass and strength decrease with aging because of muscle atrophy, eventually lowering the quality of life^29^. The application of NMN in vivo has been shown to ameliorate muscle decline in rodent models^3,4^. NMN has also been reported to improve mitochondrial functions in the skeletal muscles of rodents^3,4,30^. In agreement with the evidence reported in rodents, we found that chronic NMN supplementation partly improved muscle strength and performance in older men, which was evaluated using gait speed and grip strength; however, further investigation is needed to determine the difference between the effects of NMN on the left- and right-side grip strengths. Gait speed and grip strength are included in the diagnostic criteria for sarcopenia (aging-related loss of muscle mass and function), such as AWGS (Asian Working Group for Sarcopenia) or EWGSOP (European Working Group on Sarcopenia in Older People), and are known to be adequately sensitive for the assessment of muscle strength and performance in older adults. Therefore, we believe that the chronic oral administration of NMN is a potential therapeutic strategy for sarcopenia. Moreover, we speculate the grip strength with significance only on the left side might make a significant difference on the other side, if there are more participants. Since ~90% of Japanese people are right-handed, the effect of NMN supplementation might be more pronounced in the left hand, which is less affected by daily exercise and movement.

Contrary to the findings of the animal study, in which 300 mg/kg/day NMN tended to increase lean mass, as compared to that in the controls^9^, NMN did not affect the skeletal muscle mass of participants in our study. To examine whether our sample size was adequately large for detecting the significance of the primary endpoint, SMI, the statistical power was calculated post hoc. The standard deviation for the change in SMI at 12 weeks was 0.22 kg/m^2^ (Table 2). The value for an expected change in SMI owing to NMN intake was set at 0.45 kg/m^2^ (6–7% of baseline), based on a preliminary study on Japanese older men^31,32^. Based on these parameters, the power was calculated to be 0.991 using a two-sided test (α = 0.05 and total sample size = 20). These results indicate that the data had adequate power to determine the expected effect of NMN on SMI, even when considering the exclusion of data for 22 participants on the 12-week visit, and our data clearly rejected the hypothesis that the true effect size of NMN on SMI is 0.45 kg/m^2^ or more.

Since amino acid supplementation for 12 weeks was adequate for improving muscle mass and strength in some reports^33,34^, the period of our study was not too short for reporting muscle hypertrophy, although we cannot reject the possibility that the change in skeletal muscle mass would be observed more clearly if the experiment extended beyond 12 weeks. Recently, several NR human studies have reported that the mitochondrial functions in skeletal muscle cells do not increase following NR supplementation^16,17,19^. While our findings suggest that the chronic supplementation of NMN may support overall muscle health, further studies are warranted to elucidate the mechanisms underlying the observed increase in mobility.

NMN supplementation has also been suggested to improve insulin sensitivity and metabolic health in rodent models^3,4^. A previous NR human study reported a decrease in hepatic lipid content in men with obesity, although the decrease was not significant^13^. Furthermore, the recent study on NMN has reported insulin-stimulated glucose disposal, assessed using an hyperinsulinemic-euglycemic clamp, and increased skeletal muscle insulin signaling in response to NMN supplementation^21^. In this study, NMN exerted no effect on hepatic lipid accumulation and insulin sensitivity. This may be attributed to the normal metabolic status of our study population.

In this study, we also performed a preliminary evaluation of the auditory capacity of participants using an audiometer before and after the intervention with NMN. In mice, SIRT3, an NAD+-dependent protein deacetylase localized to the mitochondria, is reportedly involved in the regulation of hearing ability during aging^35^. NR supplementation in rodents has also been reported to improve noise-induced and age-related hearing loss via SIRT3 activation^35–37^. In our study, NMN supplementation partly tended to improve the auditory capacity of older people. However, there is limited information about the underlying mechanisms by which NAD + precursors may improve hearing in humans. Based on findings from preclinical studies^35–38^, NMN could similarly affect hearing in humans through mechanisms involving SIRT3 activation and the increased reduced-to-oxidized glutathione ratio in the mitochondria. However, in future, mechanistic studies are needed to test this hypothesis. Such studies will be technically challenging in humans, and it will be important to dissociate the effects of SIRT3 activation from the possible pleiotropic effects of the elevation of NAD+ metabolites.

While this study offers novel insights into NMN as a nutritional supplement and potential therapeutic entity, it has some limitations. First, the 42 enrolled participants were randomized between the two treatment groups that were adjusted for age, body mass index (BMI), and SMI. However, data from 22 participants were excluded, owing to which the adjustment between the two groups was disrupted, which may have compromised some results in this study. Further investigation will be needed to apply our findings to all older men, although the statistical analyses were valid for the population analyzed in this trial. Second, as all analyses were exploratory, and the primary analysis for each endpoint was specified, multiple comparisons were performed only without *P* value correction. Although the statistical analyses performed were valid for the population analyzed in this trial, further investigation is needed to confirm our findings. Third, we included only healthy older men in this study. Older participants were included in the study because NAD+ levels decrease with age in rodents and humans^39^, and NAD+ supplementation can be more effective in older people. Moreover, only male participants were selected, considering the possibility that data from older females may be affected by the rapid decrease in estrogen or progesterone levels associated with menopause. We speculate that NMN administration might be effective in different populations, such as middle-aged adults or older women, because the apparent difference in the response to NR, another NAD+ precursor, owing to age or sex, has not been reported in human clinical studies^9–19^. However, it remains to be determined whether NMN supplementation is effective in populations that are heterogeneous with respect to gender, age, or baseline physiological functions, which is critical in determining the therapeutic potential of oral NMN supplementation. Thus, further clinical studies should be conducted in specific populations in this regard.

## Methods

### Ethical approval, informed consent, and study location

The study was conducted in accordance with the guidelines of the Declaration of Helsinki and was approved by the Graduate School of Medicine and Faculty of Medicine, The University of Tokyo Research Ethics Committee (2018013P). The study was registered at UMIN-CTR (UMIN000036321) before the patients were recruited. The participants received oral and written information before they provided written consent. The study was conducted at the Clinical Research Support Center Phase 1 Unit at the University of Tokyo Hospital.

### Study design, randomization, and intervention

The study was designed as a placebo-controlled, randomized, double-blind, parallel-group trial. Participants were examined at baseline and the 6-week and 12-week visits. After completion of the baseline investigations, participants were randomized to a 12-week supplementation of NMN or a placebo, with daily administration by a third party, C&C QUALITATIVE RESEARCH INSTITUTE INC (Tokyo, Japan); there were no significant differences in age, BMI, or SMI between the two groups (Table 1). The allocation to the NMN or placebo group was also managed by C&C QUALITATIVE RESEARCH INSTITUTE INC until the end of the study. The participants received oral supplementation of 250 mg of NMN (Mitsubishi Corporation Life Sciences Limited, Tokyo, Japan) once daily or a placebo for 12 weeks. The participants and data collectors were blinded to the treatment. Once all participants completed the study, the randomization code was released.

The primary objective of this study was to evaluate the potential benefits of NMN in increasing blood NAD + concentration and its effects on the body composition of older participants after the 12-week treatment. The secondary objective of the study was to evaluate aging-related parameters, such as muscle strength and performance, bone density, vision, and hearing ability.

### Study participants

Sixty-five healthy Japanese male volunteers were recruited in the study. The inclusion criteria were as follows: male, aged more than 65 years, BMI (in kg/m^2^) 22–28, nonsmokers, and without any active diseases. Participants with a history of treatment for malignancy, heart failure, or myocardial infarction; consuming a prescription medication and/or supplement that may affect the findings of clinical research; or with a habit of daily exercise for at least 1 h for a minimum of 6 months continuously were excluded. The participants underwent a physical examination by a physician, including routine clinical biochemistry tests, to evaluate their eligibility for the study.

During the intervention, participants were instructed not to change their lifestyle and to abstain from vitamin B3-related dietary supplements. Eventually, 20 participants completed the study, and 22 dropped out because of an error in the distribution of NMN or placebo at the 6-week visit (Fig. 1).

### Evaluation of safety, tolerability, and adherence

Participants were instructed to record any adverse event in a diary, and during each visit, they were asked about any difficulty or problem they had experienced since the previous visit. Participants were also requested to immediately report any serious adverse event during the study to the investigators. Adverse events were monitored via a blood test and by observing the participants during safety checkups at the 6- and 12-week visits. Adherence was checked using the pill count.

### Laboratory measurements

Blood was collected from the forearm of each participant at baseline and the 12-week visit. Hematological parameters, including white blood cell count, red blood cell count, hemoglobin, hematocrit level, platelet count, mean red blood cell pigment content, mean red blood cell volume, and mean red blood cell pigment concentration, were measured.

An OGTT was performed using 75 g of glucose. The blood glucose, insulin, and C-peptide levels were measured at 0, 30, 60, and 120 min after oral glucose loading. The AUCs for glucose, insulin, and C-peptide were calculated using the trapezoidal formula. Insulin resistance, determined using HOMA-IR, was calculated using the following equation: fasting glucose (mg/dL) × fasting insulin (µU/mL)/405. HOMA-β was calculated using the following formula: 360 × fasting insulin (µU/mL)/(fasting glucose (mg/dL) − 63).

The levels of biochemical parameters, including triglyceride, total cholesterol, LDL-cholesterol, HDL-cholesterol, glucose, HbA1c, insulin, blood C-peptide, AST, ALT, γ-GTP, CK, total protein, albumin, uric acid, uric acid nitrogen, creatinine, sodium, potassium, high-sensitivity C-reactive protein, adiponectin, and IL-6, were measured.

For adiponectin and IL-6, blood samples were left to stand for 30 min, centrifuged at 25 °C and 1800×*g* for 5 min, and stored at −30 °C. Blood samples for adiponectin and IL-6 were dispatched to SRL, Inc. (Tokyo, Japan) for testing. Other blood tests were performed at the University of Tokyo Hospital.

### Extraction of NAD+ and LC-MS analysis

At baseline and the 12-week visit, blood samples were collected in heparinized tubes, frozen at −80 °C, and analyzed at the University of Toyama^40^. Metabolites were extracted by mixing 50 µL of blood and 450 µL of MeOH, which was followed by vortexing for 10 s. An equal volume of chloroform was added to the solution. The mixture was centrifuged at 13,000×*g* at 4 °C for 10 min. The separated upper aqueous phase was transferred into a new tube, and the same procedure was repeated. The aqueous phase was dried and reconstituted in LC/MS-grade water. Metabolites were analyzed using an Agilent 6460 Triple Quad mass spectrometer (Agilent Technologies Inc., Santa Clara, CA, USA) coupled with an Agilent 1290 HPLC system (Agilent Technologies Inc.). Analytes were separated on an Atlantis T3 column (2.1 × 150 mm, particle size 3 µm, Waters) using mobile phase A (5 mM ammonium formate) and mobile phase B (methanol) with a flow rate of 150 µL/min and column temperature of 40 °C. The programmed mobile phase gradient was as follows: 0–10 min, 0%–70% B; 10–15 min, 70% B; 15–20 min, 0% B. Data were analyzed using the MassHunter Quantitative Analysis software (Agilent Technologies Inc). A standard curve was obtained using various concentrations of the standard compounds and used for quantification.

### Body composition

A direct segmental multifrequency bioelectrical impedance analyzer (InBody S10^®^; InBody Japan Inc., Tokyo, Japan) was used to determine the body composition of the participants. We recorded the whole-body skeletal muscle mass, segmental lean (right arm, left arm, trunk, right leg, and right leg), fat mass, and body fat percentage at baseline, 6 weeks, and 12 weeks. SMI was calculated by dividing the whole-body skeletal muscle mass by the square of height (kg/m^2^).

### CT

Abdominal CT was performed to assess the liver and visceral fat (Aquilion PRIME/TSX-303A/BI, Aquilion Precison/TSX-304A/2A, Aquilion ONE/TSX-101A Vision Edition) at baseline and at the 12-week visit.

The ratio of the CT values of the liver and spleen (L/S ratio) was evaluated to assess liver fat using the images on a Centricity RA1000 workstation (GE Healthcare, Chicago, IL, USA). Three circular or ovoid regions of interest (ROIs) (diameter, ~15 mm) in the liver were placed on the left lobe and ventral and dorsal parts of the right lobe at the level of the umbilical portion of the portal vein. In contrast, two ROIs in the spleen were placed on the ventral and dorsal parts of the spleen at its maximum diameter. The apparent main vasculature, bile duct, and calcification were avoided when the ROIs were placed in each image set. The CT values and standard deviations (i.e., image noise) were recorded after placing the ROIs on each image. The CT value (L/S) was calculated as the ratio of the mean CT values of three ROIs in the liver (CT[L]) to that of two ROIs in the spleen (CT[S]).

Visceral fat was assessed using Fat Scan (East Japan Institute of Technology Co., Ltd. Ibaraki, Japan). The visceral fat area was measured in the slice at the umbilicus (Fig. 3)^41^.

### Assessment of exercise capacity and physical function

To evaluate physical functions, the participants were tested for gait speed, grip strength, and 30-s chair-stand test at baseline and at the 6- and 12-week visits.

Gait speed was assessed using a 10 m walk test with 3 m provided for acceleration/deceleration. While a participant walked about 16 m, the time was measured for walking the intermediate 10 m. The average of two measurements was used as the outcome data.

Grip strength was measured using a Smedley-type digital hand dynamometer (Grip D^®^; Matsuyoshi & Co., Ltd., Tokyo, Japan). Measurements were repeated twice for each hand. The highest handgrip strength value was used for calculations.

The 30-s chair-stand test measures the number of times a participant stood up and sitting down from the standard chair without armrests in 30 s. Participants were initially seated on the chair with their back in an upright position and were instructed to look straight forward and to rise with their arms folded.

### Hearing tests

The hearing ability of both ears was measured using an audiometer (Audiometer AA-79, RION Co., Ltd.) at baseline and at the 12-week visit. In the hearing test, only air conduction was measured, and the pure tone hearing level averages of 500, 1000×2, and 2000 Hz were evaluated.

### Cognitive function test

MMSE-J and MOCA-J were performed to assess the cognitive performance of the participants at the beginning of the study and after the 12-week intervention^42,43^.

### Flow-mediated dilation

After 10 min of rest, a blood flow-dependent vasodilatation response test was performed using a vascular ultrasound system (UNEX EF 18VG, UNEX Corporation) at baseline and at the 6- and 12-week visits. When the cuff was used to stop and release the blood flow from the forearm, the extent of blood vessel dilation was measured as the percentage of vessel diameter dilatation (lrb%).

### Statistical analysis

Statistical analysis was performed using Easy R for Microsoft Windows^44^ and R version 4.0.2 (2020-06-22) with the data collected at baseline (*N* = 21) and at the 6-week (*N* = 21) and 12-week (*N* = 10) visits in the NMN or placebo group. Outcome data are reported in terms of mean ± standard deviation. For comparisons between the NMN and placebo groups, for each outcome datum, the Shapiro–Wilk test was used as the normality test. Data that followed a normal distribution were analyzed using an unpaired *t* test. Changes in the NMN and placebo groups from baseline to Week 6 or Week 12 were compared using ANCOVA to adjust for baseline. Data that did not follow normal distribution were compared using the Mann–Whitney *U* test.

Owing to the exclusion of data in consideration of the errors in NMN or placebo allocation, treatment comparison was performed using mixed-model analysis, in which intercept and visit were included as random effects, and group, visit, and group-by-visit interaction were included as fixed effects. As some endpoints could not be calculated using mixed-model analysis, all endpoints were calculated using MMRM, in which the group, visit, and group-by-visit interaction were considered the fixed effects. The values of the outcomes at the baseline visit and covariance structure between visits was estimated without restriction. The *P* values denote group-by-visit interaction.

The primary analysis was the mixed-model analysis, or MMRM if the mixed-model analysis failed, for the endpoints measured at baseline and at the 6- and 12-week visits, and an unpaired *t* test or the Mann–Whitney *U* test was used for the endpoints measured at baseline and the 12-week visit. Statistical significance was set at *P* < 0.05 for all analyses. As all analyses were exploratory, and the primary analysis for each endpoint was specified, no correction for multiple comparisons was applied.

### Reporting summary

Further information on research design is available in the Nature Research Reporting Summary linked to this article.

## Supplementary information

Supplemental materials

Reporting Summary

## Data Availability

The datasets generated and/or analyzed during this study are available from the corresponding author on reasonable request.
