# Supplementary material for: Chronic nicotinamide mononucleotide supplementation elevates blood nicotinamide adenine dinucleotide levels and alters muscle function in healthy older men
Source: NPJ Aging. 2022 May 1;8(1):5. doi: 10.1038/s41514-022-00084-z (PMC9158788; doi:10.1038/s41514-022-00084-z)
Supplement: Supplementary file 1 — Supplemental materials [file 41514_2022_84_MOESM1_ESM.pdf]

Supplementary Table 1.
Clinical characteristics of the 20 study participants who completed the 12-week study prior to NMN supplementation, Related to Table 1.

	Placebo Mean±SD (10)	NMN Mean±SD (10)	Between group p-value
Age (year)	70.1±5.6	69.8±2.6	0.520[b]
BMI (kg/m^2)	24.8±1.2	23.7±1.3	0.063[a]
Fat Mass (%)	26.8±4.4	25.1±3.2	0.343[a]
SMI (kg/m^2)	7.79±0.44	7.65±0.39	0.459[a]
Gait Speed (m/s)	1.31±0.19	1.50±0.20	0.041[a]*
A 30-s Chair-Stand Test (Counts/30s)	13.5±5.2	14.8±4.0	0.539[a]
Right hand grip strength (kg)	36.0±7.1	40.1±3.2	0.147[b]
Left hand grip strength (kg)	34.8±4.8	36.1±5.6	0.587[a]
HbA1c (%)	5.74±0.32	5.85±0.58	0.939[b]
FBG (mg/dL)	94.4±7.8	99.0±9.0	0.236[a]
HOMA-IR	1.08±0.38	1.35±0.79	0.684[b]
CT L/S ratio	1.15±0.11	1.15±0.12	0.943[a]
Visceral adipose tissue (cm^2)	126.3±38.1	129.3±45.8	0.874[a]

The mean and standard deviation of data from 20 participants (from the NMN group (n = 10) and placebo group (n = 10)) are presented.
.
a. Inter-group comparisons were performed using an unpaired t-test.
b. Inter-group comparisons were performed using the Mann–Whitney *U* test.
*P<0.05

Supplementary Table 2.
The effect of NMN on clinical laboratory data (hematology and CRP).

	Placebo Mean±SD	NMN Mean±SD	p-value		Placebo Mean±SD	NMN Mean±SD	p-value
WBC (x10^{3}counts/μL)				RBC (x10^{3}counts/μL)			
Baseline	6.96±1.34 (21)<br>6.86±1.40 (10)	6.13±1.48 (21)<br>5.60±1.32 (10)	0.019[b]* (21:21)<br>0.069[b] (10:10)	Baseline	476.4±33.4 (21)<br>479.4±30.8 (10)	490.6±35.6 (21)<br>481.7±34.9 (10)	0.190[a] (21:21)<br>0.878[a] (10:10)
Week 12	5.61±0.78 (10)	4.85±0.87 (10)	0.054[a] (10:10)	Week 12	462.6±28.2 (10)	464.6±30.9 (10)	0.882[a] (10:10)
Change from Baseline to Week 12	-1.25±0.87 (10)	-0.75±1.20 (10)	0.416[c] (10:10)	Change from Baseline to Week 12	-16.8±19.7 (10)	-17.1±14.1 (10)	0.974[c] (10:10)
Platelet (x10^{4}counts/μL)				Hemoglobin (g/dL)			
Baseline	22.8±5.8 (21)<br>23.0±6.9 (10)	23.0±5.5 (21)<br>23.2±5.1 (10)	0.850[b] (21:21)<br>0.942[a] (10:10)	Baseline	14.7±0.9 (21)<br>14.8±0.8 (10)	15.2±0.9 (21)<br>15.1±0.7 (10)	0.091[a] (21:21).<br>0.424[a] (10:10)
Week 12	23.3±5.8 (10)	22.2±2.9 (10)	0.606[a] (10:10)	Week 12	14.4±0.5 (10)	14.5±0.7 (10)	0.555[a] (10:10)
Change from Baseline to Week 12	0.3±1.9 (10)	-1.0±3.0 (10)	0.272[a] (10:10)	Change from Baseline to Week 12	-0.4±0.6 (10)	-0.6±0.3 (10)	0.963[c] (10:10)
CRP (mg/dL)				Hematocrit (%)			
Baseline	0.115±0.162 (21)<br>0.069±0.053 (10)	0.070± 0.059 (21)<br>0.043±0.023 (10)	0.569[b] (21:21)<br>0.169[a] (10:10)	Baseline	43.8±2.3 (21)<br>43.8±2.0 (10)	44.8±2.6 (21)<br>44.4±2.5 (10)	0.170[a] (21:21)<br>0.533[a] (10:10)
Week 12	0.231±0.506 (10)	0.086±0.106 (10)	0.760[b] (10:10)	Week 12	43.0±1.5 (10)	43.0±2.1 (10)	0.990[a] (10:10)
Change from Baseline to Week 12	0.162±0.516 (10)	0.043±0.104 (10)	0.344[c] (10:10)	Change from Baseline to Week 12	-0.8±1.8 (10)	-1.4±1.3 (10)	0.558[c] (10:10)

The numbers in the parentheses indicate the respective sample sizes.

a. Inter-group comparisons were performed using an unpaired t-test. (no adjustment for baseline).
b. Inter-group comparisons were performed using the Mann–Whitney *U* test. (no adjustment for baseline).
c. Inter-group comparisons were performed using ANCOVA for adjusting the baseline
*P<0.05; **P<0.01; ***P<0.001

Supplementary Table 3.
The effect of NMN on clinical laboratory data (blood chemistry).

	Placebo Mean±SD	NMN Mean±SD	p-value		Placebo Mean±SD	NMN Mean±SD	p-value
TP (g/dL)				BUN (mg/dL)			
Baseline	7.31±0.38 (21)<br>7.32±0.40 (10)	7.46±0.31 (21)<br>7.49±0.34 (10)	0.191[a] (21:21)<br>0.318[a] (10:10)	Baseline	14.1±3.07 (21)<br>13.9±3.24 (10)	15.1±2.56 (21)<br>14.6±3.18 (10)	0.241[a] (21:21)<br>0.627[a] (10:10)
Week 12	6.90±0.29 (10)	6.98±0.34 (10)	0.576[a] (10:10)	Week 12	13.9±1.92 (10)	13.4±2.49 (10)	0.670[a] (10:10)
Change from Baseline to Week 12	-0.42±0.27 (10)	-0.51±0.22 (10)	0.779[c] (10:10)	Change from Baseline to Week 12	-0.0±2.72 (10)	-1.2±2.42 (10)	0.388[c] (10:10)
Albumin (g/dL)				Creatinine (mg/dL)			
Baseline	4.30±0.23 (21)<br>4.31±0.21 (10)	4.38±0.22 (21)<br>4.45±0.24 (10)	0.244[a] (21:21)<br>0.182[a] (10:10)	Baseline	0.913±0.12 (21)<br>0.928±0.14 (10)	0.900±0.14 (21)<br>0.865±0.15 (10)	0.687[a] (21:21)<br>0.344[a] (10:10)
Week 12	4.05±0.17 (10)	4.19±0.17 (10)	0.086[a] (10:10)	Week 12	0.950±0.16 (10)	0.870±0.16 (10)	0.253[a] (10:10)
Change from Baseline to Week 12	-0.26±0.13 (10)	-0.26±0.16 (10)	0.294[c] (10:10)	Change from Baseline to Week 12	0.021±0.05 (10)	0.000±0.03 (10)	0.372[c] (10:10)
AST (U/L)				Uric acid (mg/dL)			
Baseline	19.6±4.8 (21)<br>19.7±5.0 (10)	21.8±4.3 (21)<br>22.4±4.2 (10)	0.073[b](21:21)<br>0.210[a] (10:10)	Baseline	6.00±1.26 (21)<br>6.03±1.14 (10)	5.36±1.01 (21)<br>5.50±1.04 (10)	0.078[a] (21:21)<br>0.292[a] (10:10)
Week 12	20.1±4.5 (10)	23.8±6.3 (10)	0.148[a] (10:10)	Week 12	6.14±1.09 (10)	5.56±1.24 (10)	0.282[a] (10:10)
Change from Baseline to Week 12	0.4±2.9 (10)	1.4±4.7 (10)	0.448[c] (10:10)	Change from Baseline to Week 12	0.11±0.68 (10)	0.06±0.56 (10)	0.744[c] (10:10)
ALT (U/L)				Na (mEq/L)			
Baseline	18.0±7.7 (21)<br>18.7±10.2 (10)	21.7±8.6 (21)<br>21.8 ±7.0 (10)	0.073[b] (21:21)<br>0.139[b] (10:10)	Baseline	140.3±1.15 (21)<br>140.0±1.25 (10)	139.8±1.44 (21)<br>140.3±1.64 (10)	0.268[b] (21:21)<br>0.650[a] (10:10)
Week 12	19.1±7.4 (10)	23.6±7.6 (10)	0.197[a] (10:10)	Week 12	140.1±1.37 (10)	139.9±1.37 (10)	0.748[a] (10:10)
Change from Baseline to Week12	0.4±6.2 (10)	1.8±5.7 (10)	0.300[c] (10:10)	Change from Baseline to Week 12	0.1±0.88 (10)	-0.4±1.26 (10)	0.387[c] (10:10)
γGTP (U/L)				K (mEq/L)			
Baseline	32.4±17.2 (21)<br>31.0±18.9 (10)	31.4±16.7 (21)<br>37.1±16.6 (10)	0.840[b] (21:21)<br>0.384[b] (10:10)	Baseline	4.28±0.30 (21)<br>4.26±0.17 (10)	4.22±0.27 (21)<br>4.16±0.28 (10)	0.484[a] (21:21)<br>0.343[a] (10:10)
Week 12	36.5±32.0 (10)	36.7±13.0 (10)	0.344[b] (10:10)	Week 12	4.34±0.18 (10)	4.31±0.37 (10)	0.821[a] (10:10)
Change from Baseline to Week 12	5.5±29.9 (10)	-0.4±6.8 (10)	0.685[c] (10:10)	Change from Baseline to Week 12	0.08±0.24 (10)	0.15±0.31 (10)	0.822[c] (10:10)

The numbers in the parentheses indicate the respective sample sizes.

a. Inter-group comparisons were performed using an unpaired t-test (no adjustment for baseline).
b. Inter-group comparisons were performed using the Mann–Whitney *U* test (no adjustment for baseline).
c. Inter-group comparisons were performed using ANCOVA for adjusting the baseline

Supplementary Table 4.
The effect of NMN on whole blood NAD+ and NAD+-related metabolite levels, Related to Figure 2.

A. Baseline

	Placebo Mean±SD (n)	NMN Mean±SD (n)	Between group p-value
NMN (μM)	0.0761±0.0180 (21)<br>0.0768±0.0216 (10)	0.0750±0.0114 (21)<br>0.0795±0.0102 (10)	0.746[b] (21:21)<br>0.721[a] (10:10)
NAD+ (μM)	0.208±0.081 (21)<br>0.194±0.081 (10)	0.189±0.066 (21)<br>0.176±0.063 (10)	0.419[a] (21:21)<br>0.588[a] (10:10)
NR (μM)	0.0329±0.0115 (21)<br>0.0291±0.0036 (10)	0.0344±0.0123 (21)<br>0.0393±0.0152 (10)	0.672[b] (21:21)<br>0.105[b] (10:10)
NAMN (μM)	0.052±0.049 (21)<br>0.043±0.011 (10)	0.091±0.127 (21)<br>0.150±0.168 (10)	0.940[b] (21:21)<br>0.247[b] (10:10)
NAR (μM)	0.00073±0.00025 (21)<br>0.00066±0.00014 (10)	0.00102±0.00071(21)<br>0.00141±0.00088 (10)	0.330[b] (21:21)<br>0.009[b]** (10:10)
NA (μM)	0.00464±0.00166 (21)<br>0.00386±0.00100(10)	0.00560±0.00306 (21)<br>0.00689±0.00381 (10)	0.294[b] (21:21)<br>0.012[b]* (10:10)
NAM (μM)	15.5±2.7 (21)<br>15.9±2.6 (10)	17.5±3.2 (21)<br>17.8±3.4 (10)	0.036[a]* (21:21)<br>0.165[a] (10:10)

B. 12-week visit

	Placebo Mean±SD (n)	NMN Mean±SD (n)	Between group p-value
NMN (μM)	0.105±0.013 (10)	0.127±0.019 (10)	0.006[a]** (10:10)
NAD+ (μM)	0.53±0.12 (10)	1.07±0.16 (10)	<0.001[a]*** (10:10)
NR (μM)	0.0308±0.0088 (10)	0.0549±0.0241 (10)	0.002[b]** (10:10)
NAMN (μM)	0.05±0.03 (10)	3.51±1.86 (10)	<0.001[b]*** (10:10)
NAR (μM)	0.00073±0.00016 (10)	0.00940±0.00464 (10)	<0.001[b]*** (10:10)
NA (μM)	0.00684±0.00168 (10)	0.00974±0.00129 (10)	<0.001[a]*** (10:10)
NAM (μM)	10.6±1.6 (10)	13.4±2.4 (10)	0.006[a]** (10:10)

The numbers in the parentheses indicate the respective sample sizes.

NMN: nicotinamide mononucleotide, NAD+: nicotinamide adenine dinucleotide, NR: nicotinamide riboside, NAMN: nicotinic acid mononucleotide, NAR: nicotinic acid riboside, NA: nicotinic acid , NAM: nicotinamide

a. Inter-group comparisons were performed using an unpaired t-test.
b. Inter-group comparisons were performed using the Mann–Whitney *U* test.
*P<0.05; **P<0.01; ***P<0.001

Supplementary Table 5.
NMN supplementation does not affect metabolic parameters, Related to Figure 3.

	Placebo Mean±SD	NMN Mean±SD	p-value		Placebo Mean±SD	NMN Mean±SD	p-value
Triglycerides (mg/dL)				HDL Cholesterol (mg/dL)			
Baseline	114.6±49.5 (21)<br>102.2±44.9 (10)	116.5±42.4 (21)<br>116.7±36.8 (10)	0.897[a] (21:21)<br>0.440[a] (10:10)	Baseline	62.8±14.1 (21)<br>61.1±15.2 (10)	61.6±17.4 (21)<br>67.3±17.9 (10)	0.803[a] (21:21)<br>0.422[a] (10:10)
Week 12	82.4±36.8 (10)	107.6±37.0 (10)	0.045[b*] (10:10)	Week 12	58.7±15.2 (10)	62.1±16.4 (10)	0.635[a] (10:10)
Change from Baseline to Week 12	-19.8± 26.3 (10)	-9.1±14.2 (10)	0.133[c] (10:10)	Change from Baseline to Week 12	-2.5±4.2 (10)	-5.2±3.9 (10)	0.233[c] (10:10)
LDL Cholesterol (mg/dL)				Interleukin-6 (pg/mL)			
Baseline	134.4±26.5 (21)<br>132.2±18.0 (10)	125.7±36.8 (21)<br>125.4±32.3 (10)	0.386[a] (21:21)<br>0.568[a] (10:10)	Baseline	1.66±0.62 (21)<br>1.78±0.68 (10)	1.29±0.75 (21)<br>0.90±0.37 (10)	0.031[b*] (21:21)<br><0.001[b***](10:10)
Week 12	121.8±17.0 (10)	109.0±36.8 (10)	0.331[a] (10:10)	Week 12	1.50±0.62 (10)	1.39±0.81 (10)	0.403[b] (10:10)
Change from Baseline to Week 12	-10.4±14.5 (10)	-16.4±10.3 (10)	0.311[c] (10:10)	Change from Baseline to Week 12	-0.28±0.94 (10)	0.49±0.67 (10)	0.762[c] (10:10)
Adiponectin (μg/mL)							
Baseline	8.59±2.70 (21)<br>8.97±2.90 (10)	8.95±4.91 (21)<br>8.32±3.59 (10)	0.458[b] (21:21)<br>0.661[a] (10:10)				
Week 12	8.76±2.93 (10)	8.35±3.50 (10)	0.780[a] (10:10)				
Change from Baseline to Week 12	-0.21±0.87 (10)	0.03±1.15 (10)	0.669[c] (10:10)				

The numbers in the parentheses indicate the respective sample sizes.

a. Inter-group comparisons were performed using an unpaired t-test (no adjustment for baseline).
b. Inter-group comparisons were performed using the Mann–Whitney *U* test (no adjustment for baseline).
c. Inter-group comparisons were performed using ANCOVA for adjusting the baseline
*P<0.05

Supplementary Table 6.
The effect of NMN on auditory and cognitive functions

	Placebo Mean±SD	NMN Mean±SD	p-value		Placebo Mean±SD	NMN Mean±SD	p-value
Hearing Testing (Right/dB) p= 0.054[b]				Hearing Testing (Left/dB) p=0.636 [b]			
Baseline	23.8±12.2 (21)<br>18.1±9.7 (10)	27.2±14.0 (21)<br>25.9±10.0 (10)	0.512[d] (21:21)<br>0.095[c] (10:10)	Baseline	21.6±8.6 (21)<br>18.2±9.2 (10)	30.9±16.6 (21)<br>30.4±14.5 (10)	0.072[d]. (21:21)<br>0.045[d]* (10:10)
Week 6	24.6±13.0 (21)<br>19.3±9.6 (10)	27.6±14.3 (21)<br>26.2±10.5 (10)	0.504[d] (21:21)<br>0.146[c] (10:10)	Week 6	21.9±9.1 (21)<br>18.8±10.0 (10)	31.5±16.6 (21)<br>29.9±13.6 (10)	0.051[d]. (21:21)<br>0.045[d]* (10:10)
Week 12	20.3±10.2 (10)	25.8±9.6 (10)	0.229[c] (10:10)	Week 12	19.3±9.0 (10)	31.0±13.5 (10)	0.019[d]* (10:10)
Change from Baseline to Week 6	0.8±2.7 (21)	0.4±3.1 (21)	0.610[e] (21:21)<br>0.574[e] (10:10)	Change from Baseline to Week 6	0.3±3.8 (21)<br>0.6±2.3 (10)	0.7±3.9 (21)<br>-0.5±4.6 (10)	0.617[e] (21:21)<br>0.820[e] (10:10)
Change from Baseline to Week 12	2.1±2.7 (10)	-0.1±2.2 (10)	0.117[e] (10:10)	Change from Baseline to Week 12	1.1± 2.4 (10)	0.6±3.0 (10)	0.766[e] (10:10)
MMSE-J (score)				MOCA-J (score)			
Baseline	27.9±1.64 (21)<br>27.9±0.88 (10)	27.9±2.15 (21)<br>28.3±1.49 (10)	0.768[d] (21:21)<br>0.475[c] (10:10)	Baseline	24.9±2.49 (21)<br>25.7±1.49 (10)	24.9±2.43 (21)<br>25.7±2.54 (10)	1.000[c] (21:21)<br>1.000[c] (10:10)
Week 12	28.9±1.10 (10)	28.3±1.64 (10)	0.401[d] (10:10)	Week 12	26.0±2.00 (10)	26.2±1.48 (10)	0.802[c] (10:10)
Change from Baseline to Week 12	1.0±1.33 (10)	0.0±1.63 (10)	0.227[e] (10:10)	Change from Baseline to Week 12	0.3±2.11 (10)	0.5±2.27 (10)	0.794[e] (10:10)

The numbers in the parentheses indicate the respective sample sizes.

a. Treatment was compared using a mixed model analysis. The p-value denotes interaction.
b. Treatment was compared using MMRM. p-value denotes interaction.
c. Inter-group comparisons were performed using an unpaired t-test (no adjustment for baseline).
d. Inter-group comparisons were performed using the Mann–Whitney *U* test (no adjustment for baseline).
e. Inter-group comparisons were performed using ANCOVA for adjusting the baseline.
*P<0.05

Supplementary Table 7. The effect of NMN on BMI and vascular function

	Placebo Mean±SD	NMN Mean±SD	p-value		Placebo Mean±SD	NMN Mean±SD	p-value
BMI (kg/m2) p=0.341[a], p=0.851[b]				Systolic Blood Pressure (mmHg) p=0.982[a], p=0.438[b]			
Baseline	24.5±1.4 (21)<br>24.8±1.2(10)	24.1±1.4 (21)<br>23.7±1.3(10)	0.283[d] (21:21)<br>0.063[c] (10:10)	Baseline	130.7±19.8 (21)<br>127.0±18.2 (10)	129.5±15.3 (21)<br>135.9±18.9(10)	0.950[d] (21:21)<br>0.298[c] (10:10)
Week 6	24.4±1.3 (21)<br>24.7±1.2 (10)	24.0±1.4 (21)<br>23.6± 1.2(10)	0.283[d] (21:21)<br>0.044[c](10:10)	Week 6	126.1±19.4 (21)<br>125.2±21.3 (10)	129.2±16.1 (21)<br>136.5±20.0 (10)	0.577[c] (21:21)<br>0.237[c] (10:10)
Week 12	24.6±1.2 (10)	23.5±1.2 (10)	0.664[c] (10:10)	Week 12	128.8±20.5 (10)	131.7±25.1 (10)	0.781[c] (10:10)
Change from Baseline to Week 6	-0.1±0.3 (21)<br>-0.1±0.3(10)	-0.1±0.2 (21)<br>-0.1±0.3 (10)	0.752[e] (21:21)<br>0.377[e] (10:10)	Change from Baseline to Week 6	-4.6±12.0 (21)<br>-1.8±10.5 (10)	-0.2±7.8 (21)<br>0.6 ±7.2 (10)	0.182[e] (21:21)<br>0.584[e] (10:10)
Change from Baseline to Week 12	-0.3±0.4 (10)	-0.3±0.45 (10)	0.830[e] (10:10)	Change from Baseline to Week 12	1.8±13.0 (10)	-4.2±13.0 (10)	0.333[e] (10:10)
Flow Mediated Dilation (%) p=0.839[b]				Diastolic blood pressure(mmHg) p=0.306[b]			
Baseline	4.00±1.56 (21)<br>3.97±1.91 (10)	4.09±1.52 (21)<br>4.10±1.32 (10)	0.850[c] (21:21)<br>0.861[c] (10:10)	Baseline	79.9±9.4 (21)<br>81.2±10.9(10)	81.3±11.8 (21)<br>85.2±13.6 (10)	0.632[d] (21:21)<br>0.477[c] (10:10)
Week 6	3.80±1.93 (21)<br>4.01±2.53 (10)	3.84±1.79 (21)<br>3.25±1.76 (10)	0.941[c] (21:21)<br>0.446[c] (10:10)	Week 6	81.4±12.2 (21)<br>80.8±11.9 (10)	82.6±11.2 (21)<br>86.9±12.7 (10)	0.744[c] (21:21)<br>0.283[c] (10:10)
Week 12	3.77±1.38 (10)	3.85±1.35 (10)	0.897[c] (10:10)	Week 12	81.2±8.9 (10)	81.7±11.0 (10)	0.912[c] (10:10)
Change from Baseline to Week 6	-0.20±1.46 (21)<br>0.04±1.72 (10)	-0.24±2.25 (21)<br>-0.85±1.91 (10)	0.936[c] (21:21)<br>0.308[e] (10:10)	Change from Baseline to Week 6	1.5±7.3 (21)<br>-0.4±4.9 (10)	1.3±4.6 (21)<br>1.7±4.6 (10)	0.955[e] (21:21)<br>0.287[e] (10:10)
Change from Baseline to Week 12	-0.20±2.10 (10)	-0.25±1.72 (10)	0.925[e] (10:10)	Change from Baseline to Week 12	0.0±6.6 (10)	-3.5±8.1 (10)	0.464[e] (10:10)

The numbers in the parentheses indicate the respective sample sizes.

a. Treatment was compared using a mixed model analysis. The p-value denotes interaction.
b. Treatment was compared using MMRM. The p-value denotes interaction.
c. Inter-group comparisons were performed using an unpaired t-test (no adjustment for baseline).
d. Inter-group comparisons were performed using the Mann–Whitney *U* test (no adjustment for baseline).
e. Inter-group comparisons were performed using ANCOVA for adjusting the baseline.

Supplementary Table 1. Clinical characteristics of the 20 study participants who completed the 12-week study prior to NMN supplementation, Related to Table 1.

The mean and standard deviation of data from 20 participants (from the NMN group (n = 10) and placebo group (n = 10)) are presented.

a. Inter-group comparisons were performed using an unpaired *t*-test.

b. Inter-group comparisons were performed using the Mann–Whitney *U* test.

*P<0.05

Supplementary Table 2. The effect of NMN on clinical laboratory data (hematology and CRP).

The numbers in the parentheses indicate the respective sample sizes.

a. Inter-group comparisons were performed using an unpaired *t*-test (no adjustment for baseline).

b. Inter-group comparisons were performed using the Mann–Whitney *U* test (no adjustment for baseline).

c. Inter-group comparisons were performed using ANCOVA for adjusting the baseline.

*P<0.05

Supplementary Table 3. The effect of NMN on clinical laboratory data (blood chemistry).

The numbers in the parentheses indicate the respective sample sizes.

a. Inter-group comparisons were performed using an unpaired *t*-test (no adjustment for baseline).

b. Inter-group comparisons were performed using the Mann–Whitney *U* test (no adjustment for baseline).

c. Inter-group comparisons were performed using ANCOVA for adjusting the baseline.

*P<0.05

Supplementary Table 4.

The effect of NMN on whole blood NAD+ and NAD+-related metabolite levels, Related to Figure 2.

The numbers in the parentheses indicate the respective sample sizes.

NMN: nicotinamide mononucleotide, NAD+: nicotinamide adenine dinucleotide, NR: nicotinamide riboside, NAMN: nicotinic acid mononucleotide, NAR: nicotinic acid riboside, NA: nicotinic acid. NAM: nicotinamide

a. Inter-group comparisons were performed using an unpaired *t*-test.

b. Inter-group comparisons were performed using the Mann–Whitney *U* test.

*P<0.05; **P<0.01; ***P<0.001

Supplementary Table 5. NMN supplementation does not affect metabolic parameters, Related to Figure 3.

The numbers in the parentheses indicate the respective sample sizes.

a. Inter-group comparisons were performed using an unpaired *t*-test (no adjustment for baseline).

b. Inter-group comparisons were performed using the Mann–Whitney *U* test (no adjustment for baseline).

c. Inter-group comparisons were performed using ANCOVA for adjusting the baseline.

*P<0.05

Supplementary Table 6. The effect of NMN on auditory and cognitive functions.

The numbers in the parentheses indicate the respective sample sizes.

a. Treatment was compared using a mixed model analysis. The p-value denotes interaction.

b. Treatment was compared using MMRM. The p-value denotes interaction.

c. Inter-group comparisons were performed using an unpaired *t*-test (no adjustment for baseline).

d. Inter-group comparisons were performed using the Mann–Whitney *U* test (no adjustment for baseline).

e. Inter-group comparisons were performed using ANCOVA for adjusting the baseline.

*P<0.05

Supplementary Table 7. The effect of NMN on vascular function.

The numbers in the parentheses indicate the respective sample sizes.

a. Treatment was compared using a mixed model analysis. The p-value denotes interaction.

b. Treatment was compared using MMRM. The p-value denotes interaction.

c. Inter-group comparisons were performed using an unpaired *t*-test (no adjustment for baseline).

d. Inter-group comparisons were performed using the Mann–Whitney *U* test (no adjustment for baseline).

e. Inter-group comparisons were performed using ANCOVA for adjusting the baseline.
